# Transcriptomic characteristics of bronchoalveolar lavage fluid and peripheral blood mononuclear cells in COVID-19 patients

**DOI:** 10.1080/22221751.2020.1747363

**Published:** 2020-03-31

**Authors:** Yong Xiong, Yuan Liu, Liu Cao, Dehe Wang, Ming Guo, Ao Jiang, Dong Guo, Wenjia Hu, Jiayi Yang, Zhidong Tang, Honglong Wu, Yongquan Lin, Meiyuan Zhang, Qi Zhang, Mang Shi, Yingle Liu, Yu Zhou, Ke Lan, Yu Chen

**Affiliations:** a State Key Laboratory of Virology, Department of Infectious Disease, Zhongnan Hospital, Wuhan University, Wuhan, People’s Republic of China; b State Key Laboratory of Virology, Modern Virology Research Center, College of Life Sciences, Wuhan University, Wuhan, People’s Republic of China; c The Centre for Infection and Immunity Studies, School of Medicine, Sun Yat-sen University, Guangzhou, People’s Republic of China; d BGI PathoGenesis Pharmaceutical Technology, Shenzhen, People’s Republic of China

**Keywords:** COVID-19, transcriptome profiling, inflammation, cytokine, lymphopenia

## Abstract

Circulating in China and 158 other countries and areas, the ongoing COVID-19 outbreak has caused devastating mortality and posed a great threat to public health. However, efforts to identify effectively supportive therapeutic drugs and treatments has been hampered by our limited understanding of host immune response for this fatal disease. To characterize the transcriptional signatures of host inflammatory response to SARS-CoV-2 (HCoV-19) infection, we carried out transcriptome sequencing of the RNAs isolated from the bronchoalveolar lavage fluid (BALF) and peripheral blood mononuclear cells (PBMC) specimens of COVID-19 patients. Our results reveal distinct host inflammatory cytokine profiles to SARS-CoV-2 infection in patients, and highlight the association between COVID-19 pathogenesis and excessive cytokine release such as CCL2/MCP-1, CXCL10/IP-10, CCL3/MIP-1A, and CCL4/MIP1B. Furthermore, SARS-CoV-2 induced activation of apoptosis and P53 signalling pathway in lymphocytes may be the cause of patients’ lymphopenia. The transcriptome dataset of COVID-19 patients would be a valuable resource for clinical guidance on anti-inflammatory medication and understanding the molecular mechansims of host response.

## Introduction

The recent ongoing outbreak of viral pneumonia (COVID-19) has sparked worldwide concern. First reported in December 2019 in Wuhan, COVID-19 spread rapidly across other areas and caused a major epidemic with 80,894 confirmed cases including 3237 deaths in China by 17 March 17 2020. A novel highly infectious coronavirus, officially called the severe acute respiratory syndrome coronavirus 2 (SARS-CoV-2), was subsequently identified as the causative pathogen of this outbreak by deep sequencing and etiological investigations [1–3]. As global new cases spike in 75 more countries apart from China, WHO has declared that COVID-19 is currently a global public health emergency. According to previous studies of clinical characteristics, the most common symptoms of SARS-CoV-2 infection are fever, cough, fatigue, shortness of breath, and abnormalities on chest CT [4–6]. However, some COVID-19 patients rapidly develop severe pneumonia symptoms and complications including acute respiratory distress syndrome (ARDS), pulmonary oedema, acute kidney injury, or multiple organ failure [4–6]. The case fatality rate of COVID-19 is around 3.7% according to the national official statistics in China.

Despite increasing global threats of COVID-19, the host immune response against SARS-CoV-2 infection remains poorly understood. Viral RNAs are recognized by the innate immune system through three major classes of cytoplasmic pattern recognition receptors: Toll-like receptors (TLRs), RIG-I-like receptors (RLRs) and NOD-like receptors (NLRs), which trigger the expression of interferon (IFN) and activation of anti-viral effectors such as Natural Killer cells, T CD8+ cells and macrophages [7–10]. Coronaviruses, such as SARS-CoV and Middle East respiratory syndrome coronavirus (MERS-CoV), have evolved strategies to dampen or delay IFN production, which usually trigger exuberant inflammatory host responses leading to severe lung pathology [8,11–13]. It is believed that dysregulated host immune response and production of inflammatory cytokines, known as the “cytokine storm”, correlates with disease severity and poor prognosis during SARS-CoV and MERS-CoV infection [8,9,14]. Several proinflammatory cytokines and chemokines such as CCL-2, CCL-3, RANTES, IL-2, and IL-8 were aberrantly elevated during MERS-CoV infection of human peripheral blood monocyte-derived macrophages [8,15]. Interestingly, recent studies have reported that severe cases of COVID-19 exhibit increased plasma levels of IL2, IL6, IL7, IL10, GSCF, IP10, MCP1, MIP1A, and TNFα compared with mild cases, indicating inflammatory cytokine release is critical in COVID-19 progression [4,5]. Lymphopenia was common in SARS-CoV-2 infected patients, probably due to lymphocytes induced apoptosis [4,5,16]. Furthermore, clinical pathological analysis of COVID-19 biopsy samples confirmed interstitial mononuclear inflammatory infiltrates in lung tissues [17]. However, the underlying molecular mechanisms of the aberrant inflammatory responses in serology and histopathology under SARS-CoV-2 infection are still not clear.

Transcriptomic analyses of cells upon virus infection are extremely useful to identify the host immune response dynamics and gene regulatory networks [18,19]. In this study, we use RNA sequencing techniques to investigate the transcriptional changes in BALF and PBMC specimens of COVID-19 patients. Through transcriptomic analyses of these datasets, we identified several immune pathways and pro-inflammatory cytokines induced by SARS-CoV-2 infection, notably CCL2, CXCL2, CCL8, CXCL1, IL33, CCL3L1 in BALF and CXCL10, TNFSF10, TIMP1, C5, IL18, AREG, NRG1, IL10 in PBMC, evidencing a sustained inflammation and cytokine storm in the patients. KEGG pathway analysis of PBMC transcriptome revealed that patient’s lymphopenia may be caused by activation of apoptosis and P53 signaling pathway in lymphocytes. Altogether, our data suggest that SARS-CoV-2 infection-induced excessive cytokine release correlates with lung tissue injury and COVID-19 pathogenesis.

## Materials and methods

### Ethics statement

This study was approved by the Ethics Committee of the Zhongnan Hospital of Wuhan University. The RNA-seq analyses of BALF samples and PBMC were performed on existing samples collected during standard diagnostic tests, posing no extra burden to patients.

### Preparation of PBMC

Whole blood and PBMC (Ficoll preparation) were obtained from 3 SARS-CoV-2 patients and 3 healthy donors at Zhongnan Hospital of Wuhan University. Peripheral blood sample (4 ml) from each patient and healthy control was drawn into vacutainer tubes. The Ficoll density gradient centrifugation method was used to separate the PBMC. We diluted the blood with 1× phosphate-buffered saline (PBS) 1:1 and then transferred it to the ficoll tube. After centrifugation (20 min, 1000× g, and room temperature), the buffy coat of PBMC cells was pooled and transferred into a 15-ml falcon. PBMC was then washed twice with 10 ml PBS and centrifuged at 250× g for 10 minutes. The precipitate was collected and the total RNA was extracted.

### Preparation of BALF

2% lidocaine was injected into the segment of the lung for local anesthesia. 100 ml fractions of room temperature sterile saline were instilled into the right middle lobe or the left lingular segment of the lung. BALF was retrieved by gentle syringe suction and put into sterile containers.

### RNA isolation

Total RNA was isolated from cells and fluid with TRIzol and TRIzol LS reagents respectively under the instruction of the manufacturer.

### RNA-seq library construction and sequencing

One microgram of total RNAs were used as input. Messenger RNAs were purified using oligo-dTs covalently coupled magnetic beads. Then, the RNAs were fragmented into small pieces by heating. The expected size was 180 nt to 250 nt. First-strand of cDNA was synthesized in the presence of specific chemicals to ensure that only RNAs were used as template. Double strand cDNAs were purified with Agencourt AMPure XP beads after the reaction. DNA library was constructed through end-repair, adaptor-ligation and PCR amplification. The intermediate products were size-selected after the adaptor-ligation using two rounds of Agencourt AMPure XP beads. Qualified double-strand DNA library was transformed into single-stranded circular DNA library through DNA-denaturation and circularization. DNA nanoballs (DNBs) were generated from single-stranded circular DNA using rolling circle amplification (RCA). The DNBs were qualified using Qubit 2.0. Qualified DNBs were loaded on the flow cell and sequenced with MGISEQ-2000 platform (MGI, Shenzhen, P. R. China) for 4 PBMC samples (P1-3 and N1). Another two PBMC healthy samples (N2 and N3) were sequenced with Illumina NovaSeq platform. The RNA-seq data for BALF healthy control samples (Ctrl1, Ctrl2, and Ctrl3) were downloaded from the NCBI SRA database with accession numbers: SRR10571724, SRR10571730, and SRR10571732 [20].

### Data analysis

RNA-seq reads were firstly mapped to rRNA sequences to remove potential rRNA reads using STAR (v2.7.2b) [21] with the default parameter. The rest reads were then mapped to the human genome (hg38) with GENCODE gene annotation (v32) with parameters “–sjdbScore 1 –outFilterMultimapNmax 20 –outFilterMismatchNmax 999 –outFilterMismatchNoverReadLmax 0.04 –alignIntronMin 20 –alignIntronMax 1000000 –alignMatesGapMax 1000000 –alignSJoverhangMin 8 –alignSJDBoverhangMin 1” following the guideline of ENCODE RNA-seq pipeline (https://github.com/ENCODE-DCC/long-rna-seq-pipeline). The human un-mappable reads were then mapped to the SARS-CoV-2 genome (GenBank MN988668) as previously reported [3]. PCR replicates mapped in the human genome were removed with picard MarkDuplicates program (v2.13.2-1) [22]. RNA-seq signal tracks were generated by using bam2wig.py provided in RSeQC package (v3.0.0) [23], and visualized in UCSC genome browser [24] with custom track hub.

Gene expression was calculated by featureCounts in SubReads package (v1.5.3) [25] with the “-M” parameter. Differentially expressed genes were called by using DESeq2 package (v1.26.0) [26] with the following criteria: adjusted *p*-value < 0.05 and fold-change > 2. The expressed genes (requiring reads counts greater than 10 and 100 for BALF and PBMC, respectively), were selected and normalized to counts per millions (CPM) for further analysis.

Functional enrichment analysis was performed on the list of differentially expressed genes by using the clusterProfiler package (v 3.14.3) [27] to determine if the genes are enriched for specific terms. The level of significance for the enrichment was calculated by a hypergeometric test for each term using all expressed genes as background. The *p*-values were corrected for multiple hypothesis tests using the Benjamini and Hochberg method to control the false discovery rate (FDR). To inspect genes in specific GO-term and pathway, the GO and KEGG pathway annotation were downloaded from Gene Ontology Resource (Last updated: 22 February 2020) [28] and KEGG database (Last updated: 14 January 2020) [29], respectively. The interacting proteins to the differentially expressed genes in PBMC or BALF were identified from the functional human network InWeb_InBipMap (a human protein–protein interaction network with direct interaction evidence in high-confidence). Cytoscape (3.5.0) was used for visualizing the PPI subnetwork.

The source codes for the analysis are available at the https://github.com/zhouyulab/ncov/.

## Results

### Quantitative transcriptome analysis of BALF and PBMC from patients

**Figure 1.**
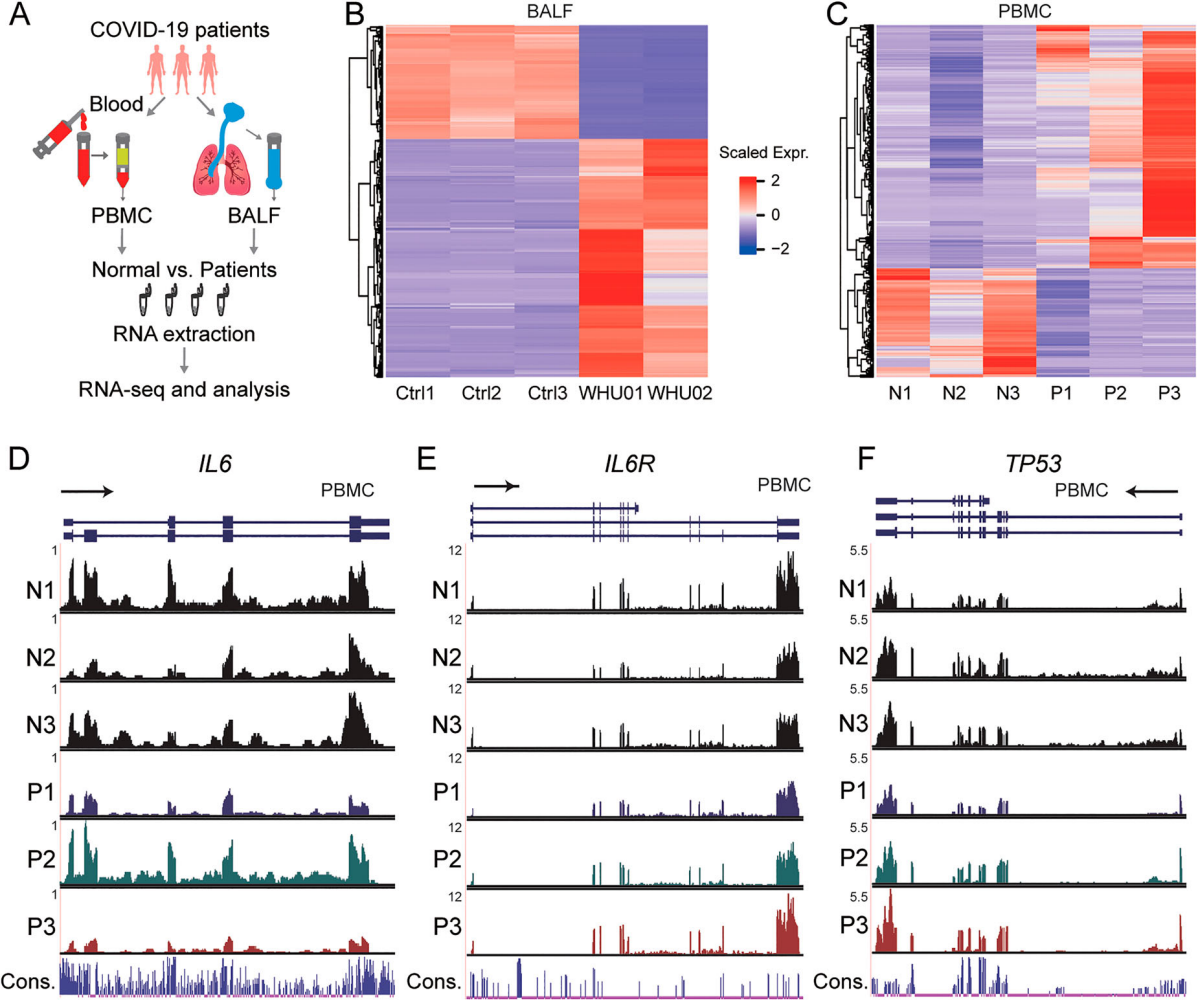
Genome-wide profiling of gene expression in BALF and PBMC of COVID-19 patients. (A) Experimental design. PBMC and BALF were prepared from patients or control. Total RNA was extracted and analysed by RNA-seq to identify differentially expressed genes implicated in COVID-19 disease pathogenesis. (B, C) Heat map of genes significantly up-regulated and down-regulated (fold change > 2) in COVID-19 patients BALF (B, WHU01-2 vs. Ctrl1-3) and PBMC (C, P1-3 vs. N1-3) compared to controls, respectively. (D-F) RNA-seq signals in PBMC patients (P1, P2, P3) and healthy controls (N1, N2, N3) for 3 genes: IL6 (D), IL6R (E), and TP53 (F), respectively. The scale on the y axis indicates the read density per million of total normalized reads.

To investigate the impact and mechanism of SARS-CoV-2 infections in patients, we exploited RNA-seq to detect transcriptome changes in both PMBC and BALF samples from healthy donors and COVID-19 patients (Figure 1(A)). We got samples from Zhongnan Hospital of Wuhan University, including BALF samples from two patients (WHU01-2), and blood samples from 3 patients (P1-3) and 3 healthy individuals (N1-3). The data for 3 BALF healthy samples (Ctrl1-3) were from a previous study [20]. We utilized Ficoll density gradient centrifugation to isolate PBMC in the blood, followed by RNA library construction and high-throughput sequencing.

We performed quality control analysis on the RNA-seq data, and summarized the statistics of reads mapped to the human genome and the SARS-CoV-2 genome (GenBank MN988668) in the Supplementary Tables 1 and 2 for BALF and PBMC samples, respectively. Consistent with our previous report focusing on viral genome assembly [3], BALF patient samples contain high ratio of viral reads, while healthy control datasets have zero counts. Interestingly, PBMC samples from patients barely exhibit viral reads, indicating that SARS-CoV-2 may not infect PBMC. The data illustrated high consistency within control or patients groups as demonstrated by the correlation and clustering analysis of BALF and PBMC samples, respectively (Supplementary Fig S1A and S1B).

For BALF samples, we detected 9609 expressed genes, in which 679 genes are up-regulated and the 325 genes are decreased in patients compared with healthy individuals (Supplementary Fig S2A). The differentially expressed genes are represented in scaled heatmap comparing patients to healthy controls for both BALF (Figure 1(B) and Supplementary File 1) and PBMC samples (Figure 1(C) and Supplementary File 1). For PBMC samples, we found more genes (15,726) with RNA expression signals, in which 707 genes are significantly up-regulated while 316 genes are down-regulated (Supplementary Fig S2B). Interestingly, as illustrated by the RNA-seq signals shown in the genome browser, IL6 and IL6R are decreased in 2 out of 3 patients (Figure 1(D,E)), while TP53 expression shows an increased trend (Figure 1(F)), although they are not identified as significantly changed genes statistically.

In sum, our data provide a global and quantitative resource for investigating the RNA regulation upon SARS-CoV-2 infection.

### Functional enrichment analysis of regulated genes

**Figure 2.**
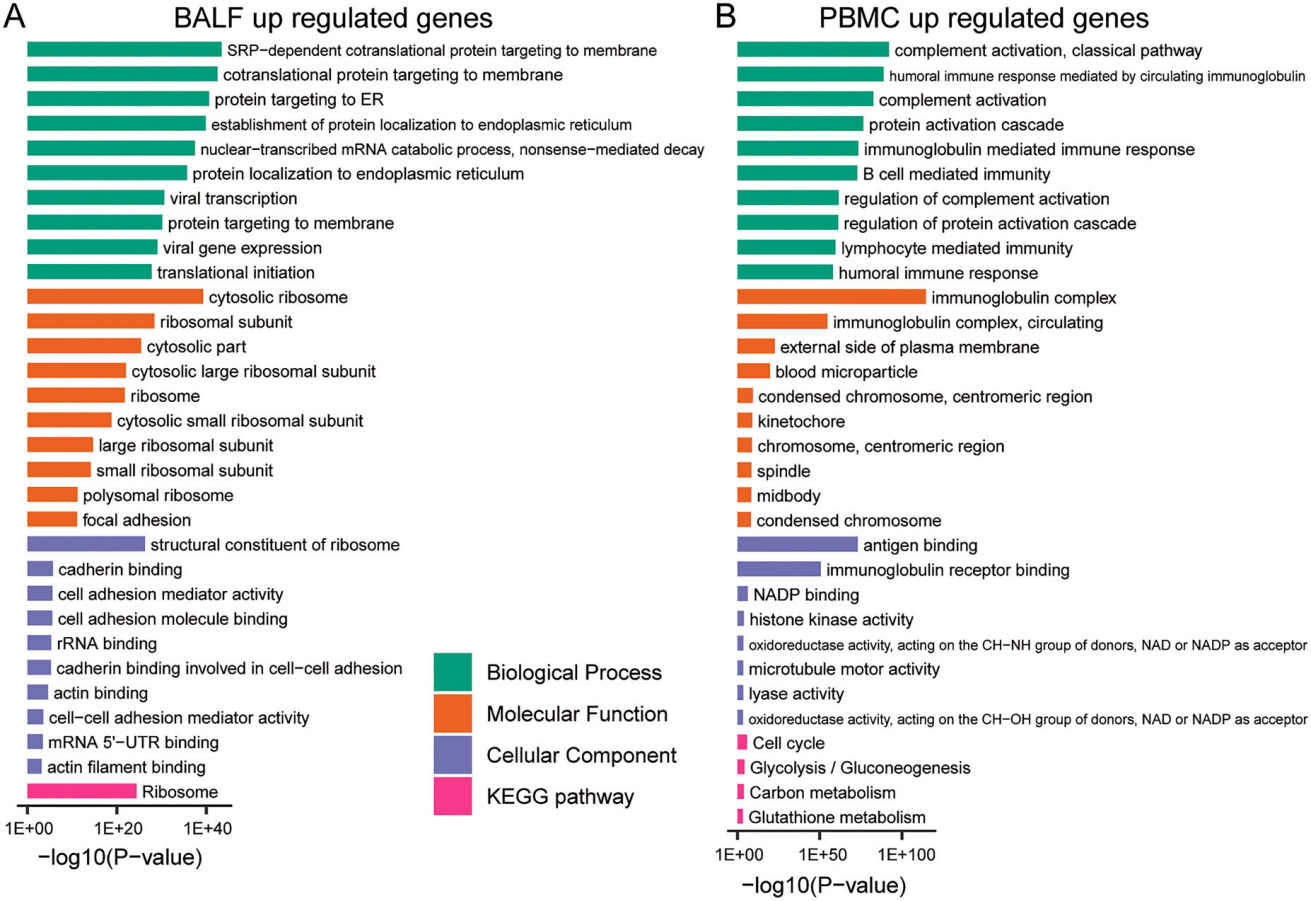
GO-term and KEGG pathway enrichment of up-regulated expressed genes in BALF and PBMC of COVID-19 patients. (A) GO-term functional enrichment by 3 categories (BP, MF, CC) and KEGG pathway analysis were performed for up-regulated genes in COVID-19 patients BALF. (B) Same as (A) for up-regulated genes in COVID-19 patients PBMC.

Virus infection may induce dynamic changes of gene expression in specific cellular biological processes. We performed gene functional enrichment analysis of the differentially regulated genes in BALF and PBMC to monitor the changes in cells of patients and healthy persons. For BALF samples, the up-regulated genes are related to invasion of the virus (Figure 2(A) and Supplementary File 2). Viral infection-induced changes in various membrane structures and endoplasmic reticulum. Indeed, the most enriched biological processes are “cotranslational protein targeting to membrane”, “protein targeting to ER”, and “viral transcription”. However, up-regulated genes in PBMC are mainly enriched in “complement activation”, “humoral immune response mediated by circulating immunoglobulin”, and “B cell mediated immunity” (Figure 2(B) and Supplementary File 2), indicating activated immune activity in PMBC. In addition, a series of inflammation-related processes was activated, such as “regulation of acute inflammatory response” and “acute inflammatory response” (Supplementary File 2).

**Figure 3.**
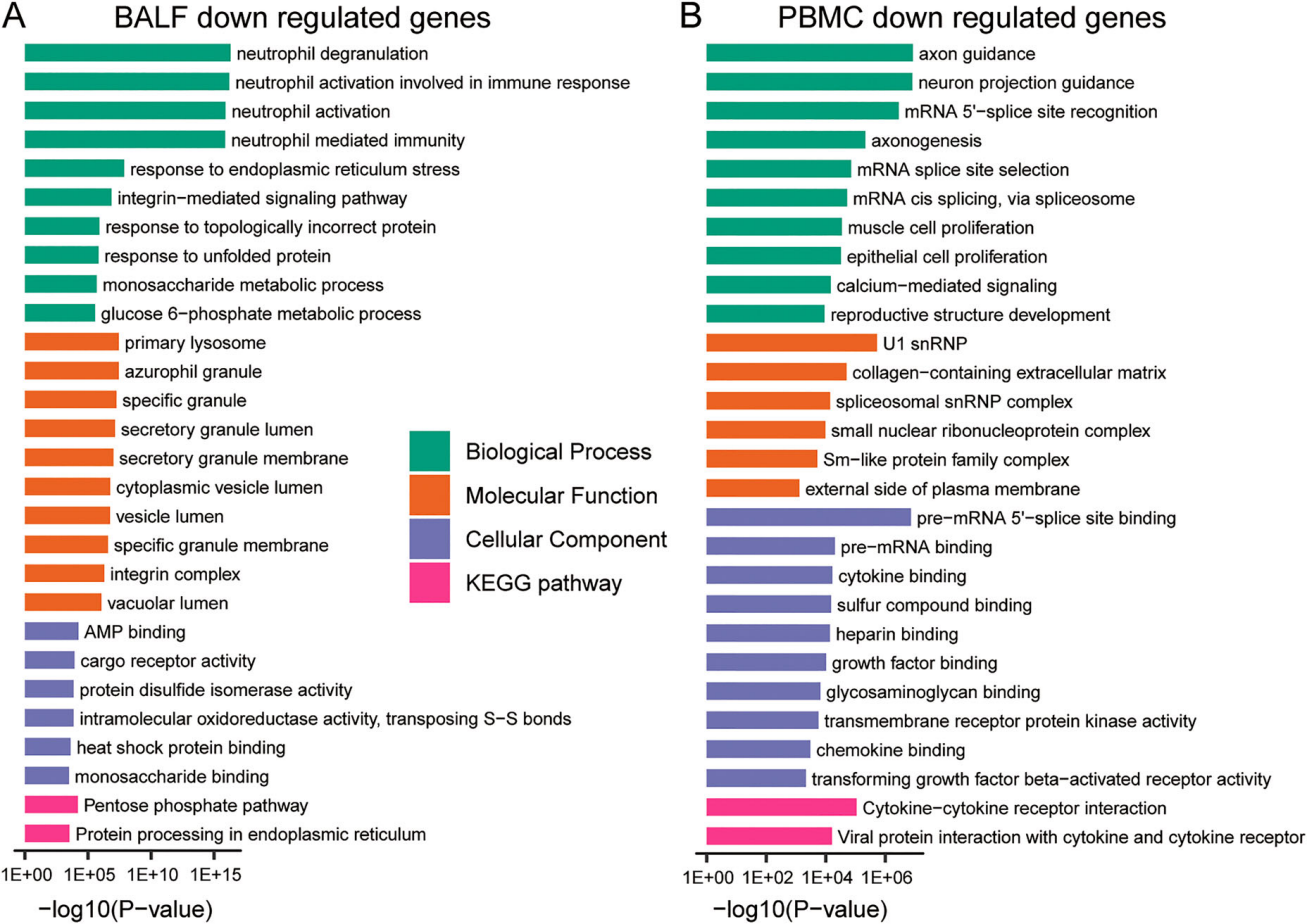
GO-term and KEGG pathway enrichment of down-regulated expressed genes in BALF and PBMC of COVID-19 patients. (A) GO-term functional enrichment by 3 categories (BP, MF, CC) and KEGG pathway analysis were performed for down-regulated genes in COVID-19 patients BALF. (B) Same as (A) for down-regulated genes in COVID-19 patients PBMC.

Interestingly, the decreased genes in BALF of patients are enriched in biological processes of “activation of immune cells” (Figure 3(A) and Supplementary File 3). In contrast, the decreased genes in PBMC of patients are involved in other biological processes such as “axon guidance”, “neuron projection guidance” and “mRNA-related biological processes” (Figure 3(B) and Supplementary File 3).

Furthermore, KEGG pathway analysis was performed for these up- and down-regulated genes. In BALF samples, the pathways enriching changed genes include “Ribosome”, “Protein processing in endoplasmic reticulum”, “Phagosome”, “Pentose phosphate pathway”, “Carbon metabolism”, and “lysosome” (Figure 2(A) and Figure 3(A), Supplementary File 2 and File 3). For PBMC samples, “Cell cycle” and “metabolism” pathways enrich up-regulated genes, while cytokine-related pathways enrich down-regulated genes, such as “Viral protein interaction with cytokine and cytokine receptor”, “NF−kappa B signaling pathway”, “Toll−like receptor signaling pathway”, and “IL−17 signaling pathway” (Figures 2 and 3(B), Supplementary File 2 and File 3). The interacting proteins to the differentially expressed genes in BALF and PBMC were also identified from the functional human network to obtain a global understanding of their interaction network (Supplementary Fig S3).

### Analysis of regulated cytokines

**Figure 4.**
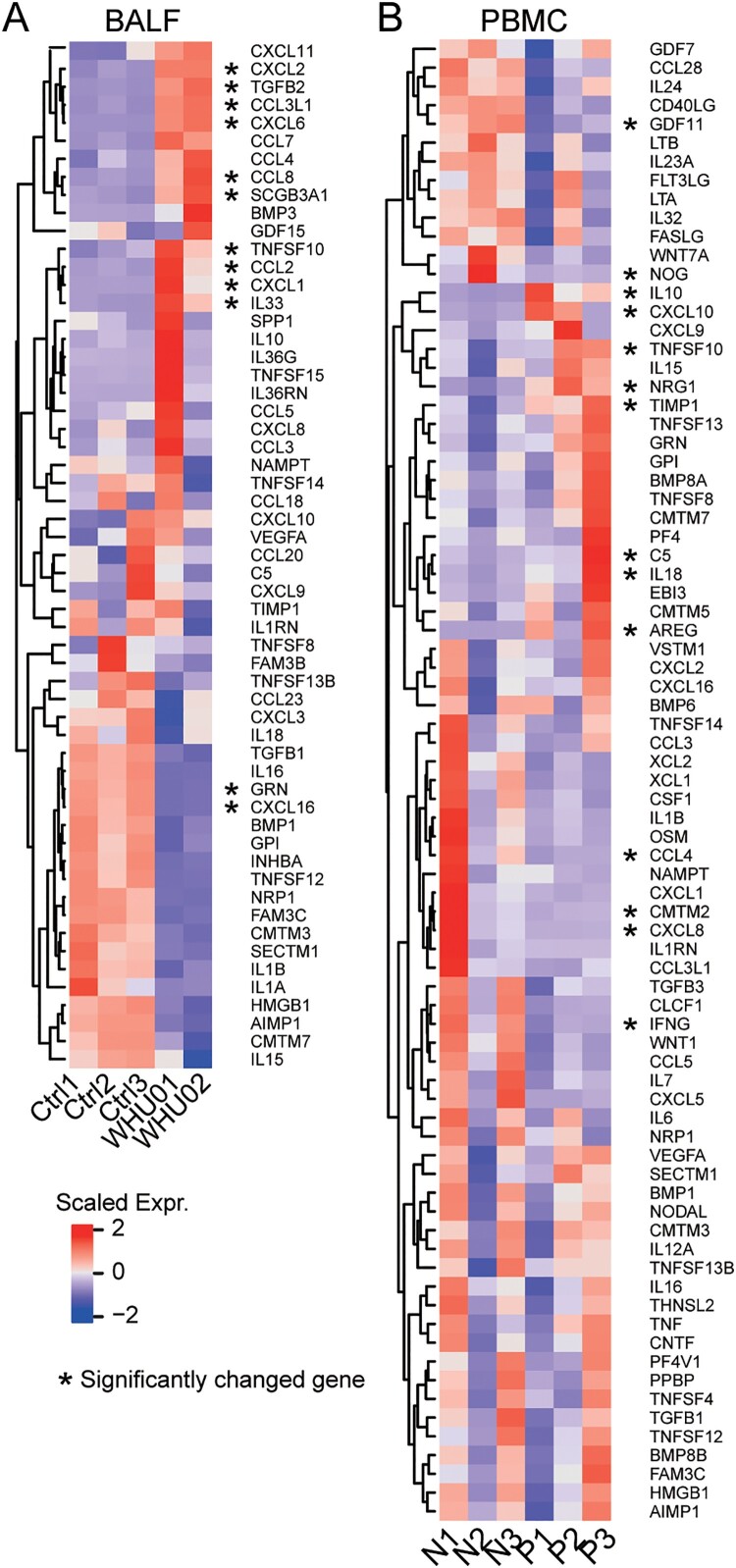
Inflammatory cytokines expression in COVID-19 patients. Heat map depicting inflammatory cytokine genes expression in COVID-19 patients BALF (A, WHU01-02 vs. Ctrl1-3) and PBMC (B, P1-3 vs. N1-3) compared with control. Genes significantly up-regulated and down-regulated are labelled with asterisks.

According to previous clinical reports, COVID-19 patients have cytokine storm which is in reminiscence of SARS-CoV infection [4,12,32]. We identified all expressed cytokines in BALF (Figure 4(A)) and PBMC (Figure 4(B)) with significantly changed genes marked by an asterisk. As in clinical cases, high cytokine expression was observed in the BALF. In agreement with laboratory findings, we found cytokines IL10, CCL2/MCP-1, CXCL10/IP-10, CCL3/MIP-1A, and CCL4/MIP1B are highly expressed in patients’ BALF samples. Further comparison between the two patients (WHU01 and WHU02) indicated the expression of some cytokines, such as IL10, IL36RN, IL36G, TNFSF15, CCL5, TNFSF10, CXCL1, and IL33, are variable among patients.

### SARS-CoV-2 infection may cause lymphocyte apoptosis

**Figure 5.**
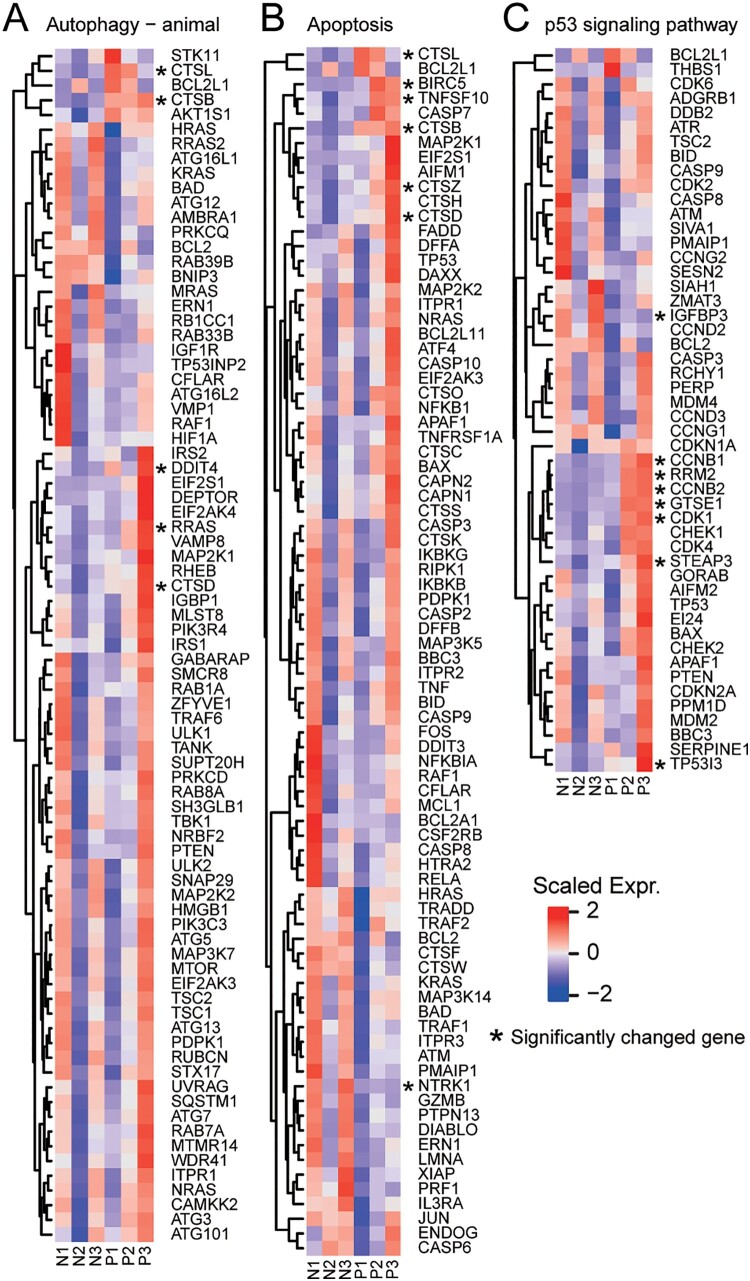
Apoptosis-related pathway in PBMC. The heatmaps show the expression levels of differentially expressed genes in different signaling pathways, including (A) autophagy (- animal species) signal pathway, (B) apoptosis signal pathway, (C) p53 signaling pathway. Genes significantly up-regulated and down-regulated are labelled with asterisks.

Laboratory findings of the 3 patients (Supplementary Table 3) indicated cell count reduction of various types of immune cells including lymphocytes in patients’ blood. Previous clinical and autopsy reports indicated that patients’ lymphocytes have been greatly reduced [5]. We found that several significantly altered genes are enriched to the apoptosis and P53 signaling pathways (Figure 5), including CTSL, CTSB, DDIT4, RRAS, CTSD, BIRC5, TNFSF10, CTSZ, NTRK1, IGFBP3, CCNB1, RRM2, CCNB2, GTSE1, CDK1, STEAP3, and TP53I3. Interestingly, TP53, an important gene in the process of apoptosis, shows an increasing trend in two patients, indicating that PMBCs reduction may be due to apoptosis.

### Comparing differently changed genes in BALF and PBMC from patients

**Figure 6.**
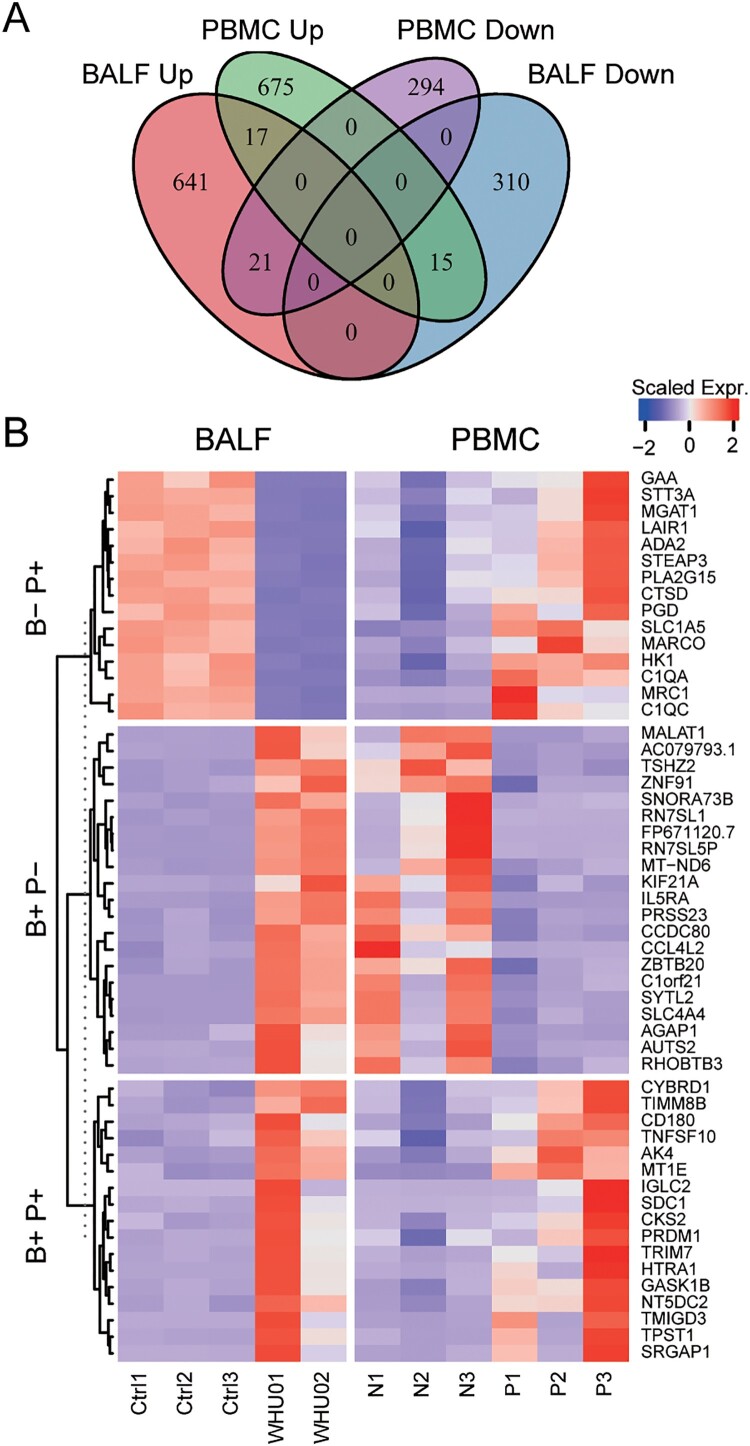
Comparison of differentially expressed genes in BALF and PBMC. (A) Venn diagram showing the number of changed genes with same or different trends between BALF and PBMC samples. (B) Heat map depicting the scaled gene expression changes with same or different trends between BALF and PBMC samples.

Based on the differential gene expression analysis, we found that there are significant differences in the levels of intracellular gene changes between the BALF samples and PBMC. This distinction is due to differences in viral infections to the two types of cells. Of note, the virus seems not to infect PBMC as that we failed to detect viral RNAs as well as ACE2 expression in PBMC (Supplementary Tables 1 and 2), which was also supported by the expression data base of bone marrow-derived blood cells (http://www.altanalyze.org). In order to illustrate the differences between the two cell types, we classified genes with different trends between the BALF samples and PBMC (Figure 6(A,B)). There are 17 genes that change in the same direction, while 36 genes have opposite trends. We first analysed genes that increased in PBMC but decreased in BLAF. Among the 15 genes, 5 genes are participated in small molecule catabolic process: ADA2, HK1, MGAT1, PGD and PLA2G15. And 4 genes are involved in neutrophil activation and immunity: ADA2, CTSD, GAA, LAIR1 (Supplementary File 4). Next, we analysed genes that increased in BLAF but decreased in PBMC, which were listed in Figure 6(B). Although, there are 17 genes changing in the same trend between the two samples, the biological meaning still needs further investigation.

## Discussion

The infection of SARS-CoV-2 can cause severe pulmonary disease and complications with significant morbidities and mortalities. Currently, there is no optimal treatment or effective drug for this fatal lung disease. Our understanding of the host immune response to SARS-CoV-2 infection is limited, making it difficult to design novel therapeutics. Viral infection usually causes massive alterations in the host transcriptome, leading to aberrant host cell metabolism and modulated immune response which is ideal for viral replication [12,33]. Using several patients’ and healthy individuals’ BALF and PBMC samples, we performed genome-wide RNA-sequencing to characterize the host immune response in COVID-19 patients. We found that 1004 and 1023 genes were differential expressed (*p* < 0.05) when BALF and PBMC samples of COVID-19 patients were compared to control, respectively. As expected, these genes fall into categories including humoral immune response, lymphocyte mediated immunity and complement activation, which all play important roles in restricting viral infection.

Inflammatory response in viral pneumonia can be a double-edged sword. Although beneficial inflammation is necessary for the local tissues to fight infection, exacerbated inflammatory responses in pneumonia patients result in excessive release of pro-inflammatory cytokines known as “cytokine storm”, leading to detrimental outcomes such as diffuse alveolar damage and fibrosis, progressive respiratory failure and multiple organ dysfunction[4,34,35]. The newly emerged SARS-CoV-2 virus shares the same human receptor ACE2 with SARS-CoV virus [1]. One of the hallmarks of SARS disease are systemic inflammation and cytokine storm with increased level of IL-6, IL-8, CXCL10/IP-10, CCL2/MCP-1 and CCL3/MIP-1A [12,36]. Little is known about the immunopathology of COVID-19 diseases. Recent clinical investigation reveals that COVID-19 mild patients had high level of IL1B, IFNγ, CXCL10/IP-10 and CCL2/MCP-1, while patients requiring ICU admission had higher level of GCSF, CXCL10/IP-10, CCL2/MCP-1 and CCL3/MIP-1A [4]. Consistently, in this study we found the expression of a large number of cytokines are significantly elevated in COVID-19 patients BALF samples compared to control, including pro-inflammatory cytokines CXCL1, CXCL2, CXCL6, CXCL8, CXCL10/IP-10, CCL2/MCP-1, CCL3/MIP-1A and CCL4/MIP1B. These data suggest SARS-CoV-2 virus infection led to cytokine storm which correlated with disease severity. Increased transcription of respective chemokines receptors such as CCR2 (CCL2/MCP-1 receptor) and CCR5 (CCL3/MIP-1A receptor) was also observed, indicating the activation of these inflammatory signaling. Cytokines/chemokines and their receptors play an important role in the cytokine migration and activation of immune cells at the sites of infection [37]. It has been shown that CCR2 and CCR5 deficient mice infected with mouse-adapted SARS-CoV virus exhibited defects in directing inflammatory cell to the airway, causing severe disease and increased mortality [38]. In addition, our results reveal that high levels of macrophages chemoattractant CXCL10/IP-10 and CCL2/MCP-1 and neutrophil chemoattractant CXCL2 and CXCL8 facilitate the migration of these immune cells to the site of infection, which was consistent with mononuclear cell infiltrates in lung tissues of COVID-19 patients [17].

Induction of inflammatory cytokines is tightly controlled to prevent tissue damage and maintain a proper immune homeostasis. It has been shown that pro-inflammatory cytokines IL-6, which is required for the regulation of inflammatory response, B-cell differentiation and antibody production, was increased in SARS patients [39,40]. Laboratory findings indicated serum IL-6 protein level increased in 2 out of 3 patients (Supplementary Table 3). However, IL-6 transcription levels did not significantly change in PBMC (Figure 1(D) and Supplementary File 1), indicating that the secreted IL-6 protein in serum might be from lung epithelial cells. Very recently, administration of the anti-IL-6-Rα antibody *tocilizumab* has been proposed as a treatment in clinical practice by ameliorating inflammation of COVID-19 patients. Of note, we noticed that relatively lower expression levels of IL6R was observed in BALF of COVID-19 patients compared with healthy individual (Supplementary File 1), and there are no significant differences in PBMC (Figure 1(E) and Supplementary File 1), suggests that IL-6/IL6R axis of cells in BALF and PBMC might not be involved in the pathophysiology of COVID-19 disease. A more comprehensive clinical study and larger scale of transcriptome profiling with more cases is needed to evaluate the justification of anti-IL-6 mAb treatment.

Moreover, we found that anti-inflammatory cytokines IL-10 and TGF-b were also induced upon SARS-CoV-2 virus infection, resembling the high TGF-b activity found in SARS patients [34,41]. TGF-b is a multifunctional cytokine which regulated a variety of biological processes including cell proliferation, differentiation, apoptosis, and immune responses [42]. Additionally, TGF-b signaling can be modulated by virus infection to block cell apoptosis and to promote fibroblast proliferation and myofibroblast differentiation, thus plays a critical role in the development of pulmonary fibrosis [43]. Recently, COVID-19 patient’s lung biopsy has been shown mild fibrosis and moderate inflammation [17]. Our data suggests that increased expression of TGF-b in COVID-19 patients might be the cause of pulmonary fibrosis.

Lymphopenia have been found in a large portion of SARS and COVID-19 patients, although the underlying mechanism remains unclear [4,6,44–47]. Viral infection of lymphocytes was barely detected in SARS patients [48]. Similarly, we have not observed any viral gene expression in patients PBMC, indicating SARS-CoV-2 virus may not infect lymphocytes in COVID-19 patients. Among these 3 patients, autophagy, P53 and apoptosis pathways are highly expressed in PBMC of COVID-19 patients compared with healthy individual. Due to its anti-inflammatory effect, glucocorticoids have been widely used to stop the progression of the acute lung injury and acute respiratory distress syndrome in SARS and COVID-19 patients by suppressing lung inflammation [4,49]. However, glucocorticoids treatment has been criticized for its significant side effects including immunosuppression, impaired antibody responses, delayed viral clearance and more severely, avascular necrosis and osteoporosis [44,49–51]. Collectively, lymphopenia in SARS and COVID-19 patients was more likely caused by endogenous or exogenous glucocorticoids which ultimately led to apoptosis of lymphocytes, rather than direct viral infection of these cells [44,50].

In summary, the present work demonstrated that SARS-CoV-2 virus infection stimulates a unique transcriptome profile in COVID-19 patients BALF and PBMC. Additionally, the cytokine expression profile suggests excessive pro-inflammatory cytokine release might be a hallmark of COVID-19 patients. Furthermore, the COVID-19 patients BALF and PBMC RNA-seq dataset provides a useful resource to the community for further thorough analyses.

## Author Contributions

YC, KL and YZ conceptualized the study design; YX and HWJ recruited the patients, collected specimens, collected demographic and clinical data; AJ, HW, YQL and MZ constructed and sequenced the RNA-seq libraries; YL and MG did the laboratory tests; DW, JY, ZT, YZ, LC and MS analysed the sequencing data; LC, YL and YC interpreted the results; YL and LC wrote the initial drafts of the manuscript; YL, YC, YZ and KL revised the manuscript. All authors read and approved the final manuscript.

## Supplementary Material

Supplemental Material

## Data Availability

The raw sequencing data from this study have been deposited in the Genome Sequence Archive [30] in BIG Data Center (https://bigd.big.ac.cn/) [31], Beijing Institute of Genomics (BIG), Chinese Academy of Sciences, under the accession number: CRA002390.
