# Supplementary material for: Transcriptomic characteristics of bronchoalveolar lavage fluid and peripheral blood mononuclear cells in COVID-19 patients
Source: Emerg Microbes Infect. 2020 Mar 31;9(1):761–70. doi: 10.1080/22221751.2020.1747363 (PMC7170362; doi:10.1080/22221751.2020.1747363)
Supplement: Supplemental Material [file TEMI_A_1747363_SM1750.zip › SupplementaryTable1.docx]

**Supplementary Table 1. Statistics of RNA-seq reads from BALF samples**

| Sample | Raw | Trimmed | hg38 | hg38  non-dup. | hg38  unmapped | SARS-CoV-2 |
| --- | --- | --- | --- | --- | --- | --- |
| WHU01_rep1 | 8,445,319 | 8,232,657 | 6,685,947(79.17%) | 1,078,408 | 1,416,549 | 134,682(2.83%) |
| WHU01_rep2 | 3,684,510 | 3,594,893 | 2,628,309(71.33%) | 498,179 | 586,280 | 52,312(1.42%) |
| WHU02_rep1 | 4,765,003 | 4,647,978 | 3,784,942(79.43%) | 1,128,148 | 708,496 | 31,694(0.38%) |
| WHU02_rep2 | 2,261,279 | 2,209,776 | 1,583,364(70.02%) | 574,653 | 314,465 | 13,358(0.59%) |
| ctrl1 | 63,238,937 | 62,100,998 | 54,676,762(86.46%) | 36,950,832 | 577,175 | 0(0.00%) |
| ctrl2 | 61,406,004 | 60,233,445 | 54,086,464(88.08%) | 36,354,889 | 602,529 | 0(0.00%) |
| ctrl3 | 63,175,835 | 62,089,610 | 54,657,415(86.52%) | 33,100,661 | 678,846 | 0(0.00%) |
