# Supplementary material for: Transcriptomic characteristics of bronchoalveolar lavage fluid and peripheral blood mononuclear cells in COVID-19 patients
Source: Emerg Microbes Infect. 2020 Mar 31;9(1):761–70. doi: 10.1080/22221751.2020.1747363 (PMC7170362; doi:10.1080/22221751.2020.1747363)
Supplement: Supplemental Material [file TEMI_A_1747363_SM1750.zip › SupplementaryTable2.docx]

**Supplementary Table 2. Statistics of RNA-seq reads from PBMC samples**

| Sample | Raw | rRNA | hg38 | hg38  non-dup. | hg38  unmapped | SARS-CoV-2 |
| --- | --- | --- | --- | --- | --- | --- |
| N1 | 78,223,959 | 44,020 | 72,356,720(92.50%) | 72,356,720 | 2,065,623 | 0(0.00%) |
| N2 | 48,579,245 | 2,523,539 | 34,794,549(71.62%) | 34,794,549 | 975,025 | 0(0.00%) |
| N3 | 48,791,891 | 5,606,164‬ | 34,874,151(71.48%) | 34,874,151 | 1,224,322 | 0(0.00%) |
| P1 | 76,591,635 | 59,446 | 62,817,957(82.02%) | 62,817,957 | 1,359,901 | 2(0.00%) |
| P2 | 76,523,698 | 35,658 | 67,004,854(87.56%) | 67,004,854 | 1,641,955 | 0(0.00%) |
| P3 | 78,648,462 | 40,521 | 72,718,082(92.46%) | 72,718,082 | 1,803,081 | 0(0.00%) |
