# Supplementary material for: Transcriptomic characteristics of bronchoalveolar lavage fluid and peripheral blood mononuclear cells in COVID-19 patients
Source: Emerg Microbes Infect. 2020 Mar 31;9(1):761–70. doi: 10.1080/22221751.2020.1747363 (PMC7170362; doi:10.1080/22221751.2020.1747363)
Supplement: Supplemental Material [file TEMI_A_1747363_SM1750.zip › SupplementaryTable3.docx]

**Supplementary Table 3. Clinical laboratory test on the February 12**

| **Items** | Patient1 | Patient2 | Patient3 | Normal range of  Lab text |
| --- | --- | --- | --- | --- |
| **Age** | 74 | 37 | 25 |  |
| [**Gender**](C:/%E5%BA%94%E7%94%A8%E7%A8%8B%E5%BA%8F/Dict/8.8.1.0/resultui/html/index.html#/javascript:;) | Male | Male | Male |  |
| **WBC** | 5.58×10^9/L | 3.47×10^9/L↓ | 11.57×10^9/L↑ | 3.5-9.5×10^9/L |
| **RBC** | 2.92×10^12/L↓ | 2.39×10^12/L↓ | 4.61×10^12/L | 4.3-5.8×10^9/L |
| **Hb** | 85.2g/L↓ | 97.1g/L↓ | 140.6g/L | 130-175g/L |
| **Platelet** | 55×10^9/L↓ | 100×10^9/L | 274×10^9/L | 125-350×10^9/L |
| **Neutrophil** | 5.26×10^9/L | 2.38×10^9/L | 10.21×10^9/L↑ | 1.8-6.3×10^9/L |
| **Lymphocyte** | 0.14×10^9/L↓ | 0.64×10^9/L↓ | 0.54×10^9/L↓ | 1.1-3.2×10^9/L |
| [**Monocyte**](C:/%E5%BA%94%E7%94%A8%E7%A8%8B%E5%BA%8F/Dict/8.8.1.0/resultui/html/index.html#/javascript:;) | 0.17×10^9/L | 0.33×10^9/L | 0.81×10^9/L↑ | 0.1-0.6×10^9/L |
| **IL-6** | 48.13pg/mL↑ | 1.93pg/mL | 16.16pg/mL↑ | 0.1-2.9pg/mL |
| **IFNγ** | 0.58pg/mL | 0.11pg/mL | 0.26pg/mL | 0.1-18pg/mL |
| **IL-10** | 11.41pg/mL↑ | 6.18pg/mL↑ | 3.22pg/mL | 0.1-5pg/mL |
| **Confirmatory test done** | Yes | Yes | Yes |  |
