# Supplementary figures and images for: Transcriptomic characteristics of bronchoalveolar lavage fluid and peripheral blood mononuclear cells in COVID-19 patients
Source: Emerg Microbes Infect. 2020 Mar 31;9(1):761–70. doi: 10.1080/22221751.2020.1747363 (PMC7170362; doi:10.1080/22221751.2020.1747363)

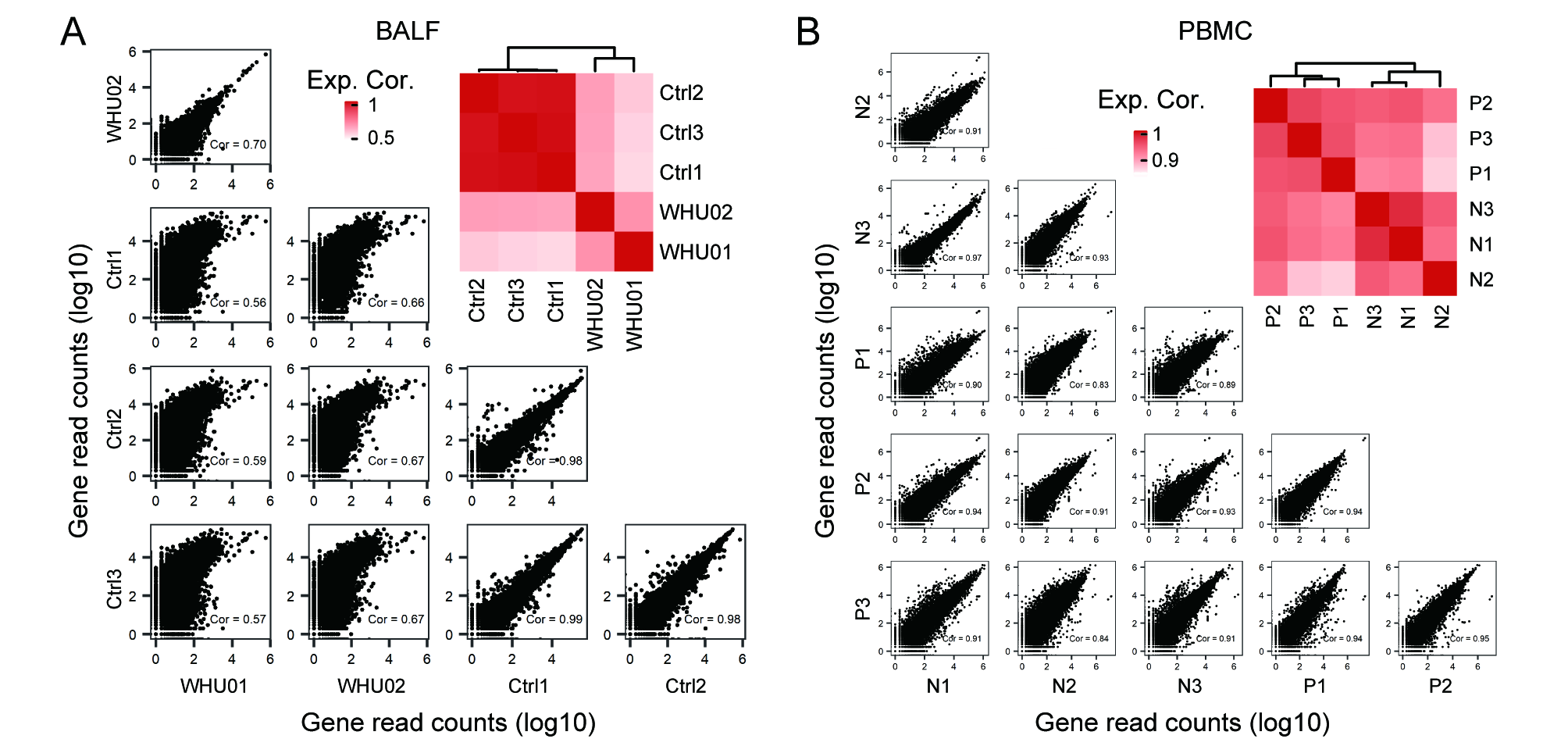

Supplement: Supplemental Material [file TEMI_A_1747363_SM1750.zip › SuppFig1.tif]

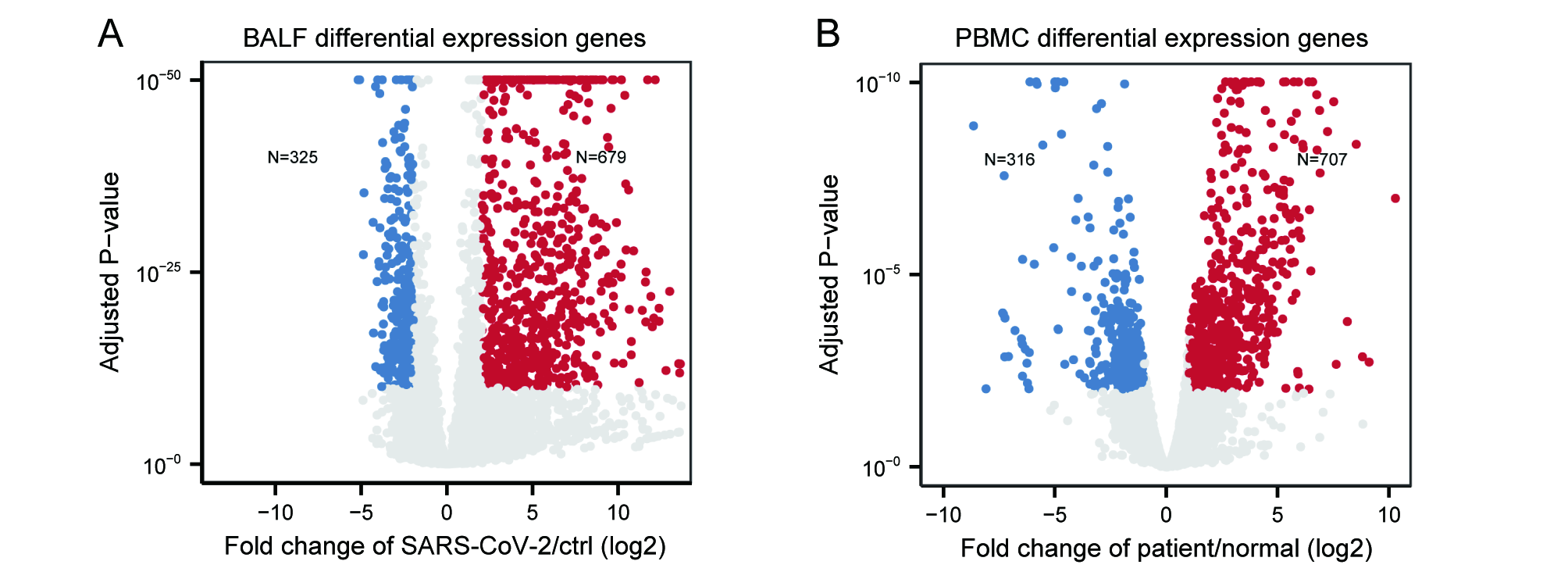

Supplement: Supplemental Material [file TEMI_A_1747363_SM1750.zip › SuppFig2.tif]

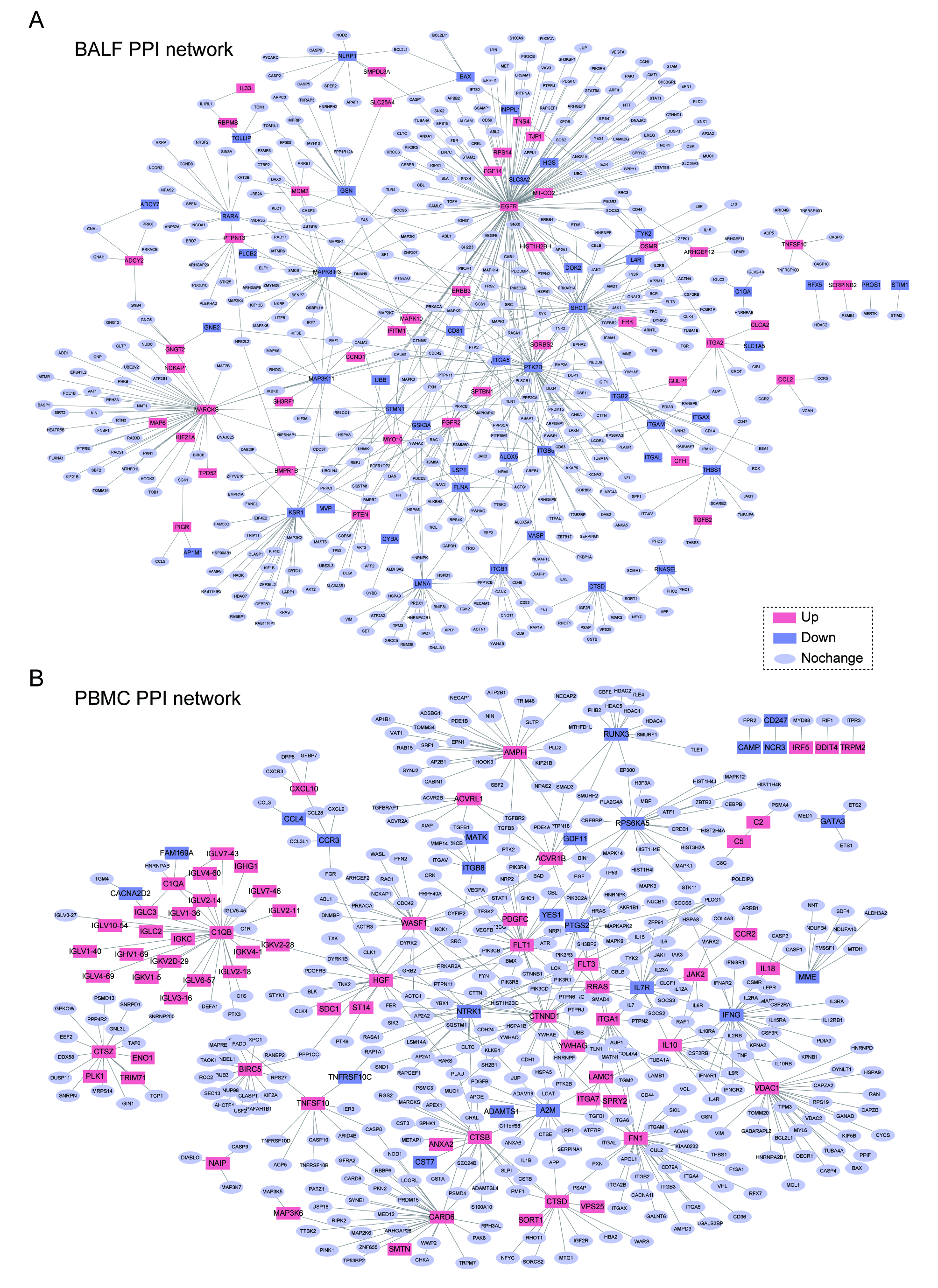

Supplement: Supplemental Material [file TEMI_A_1747363_SM1750.zip › SuppFig3.tif]
